# Genetic polymorphism of merozoite surface protein-3 in Myanmar *Plasmodium falciparum* field isolates

**DOI:** 10.1186/s12936-020-03256-y

**Published:** 2020-05-19

**Authors:** Hương Giang Lê, Thị Lam Thái, Jung-Mi Kang, Jinyoung Lee, Mya Moe, Tuấn Cường Võ, Haung Naw, Moe Kyaw Myint, Zaw Than Htun, Tong-Soo Kim, Ho-Joon Shin, Byoung-Kuk Na

**Affiliations:** 1 Department of Parasitology and Tropical Medicine, Gyeongsang National University College of Medicine, Jinju, 52727 Republic of Korea; 2 BK21Plus Team for Anti-aging Biotechnology and Industry, Department of Convergence Medical Science, Gyeongsang National University, Jinju, 52727 Republic of Korea; 3 Department of Tropical Medicine, Inha University College of Medicine, Incheon, 22212 Republic of Korea; 4 Department of Medical Research Pyin Oo Lwin Branch, Pyin Oo Lwin, Myanmar; 5 Department of Microbiology, Ajou University School of Medicine, Suwon, 16499 Republic of Korea

**Keywords:** *Plasmodium falciparum*, Merozoite surface protein-3, Genetic diversity, Natural selection, Myanmar

## Abstract

**Background:**

*Plasmodium falciparum* merozoite surface protein-3 (PfMSP-3) is a target of naturally acquired immunity against *P. falciparum* infection and is a promising vaccine candidate because of its critical role in the erythrocyte invasion of the parasite. Understanding the genetic diversity of *pfmsp*-*3* is important for recognizing genetic nature and evolutionary aspect of the gene in the natural *P. falciparum* population and for designing an effective vaccine based on the antigen.

**Methods:**

Blood samples collected from *P. falciparum*-infected patients in Naung Cho and Pyin Oo Lwin, Myanmar, in 2015 were used in this study. The *pfmsp*-*3* was amplified by polymerase chain reaction, cloned, and sequenced. Genetic polymorphism and natural selection of Myanmar *pfmsp*-*3* were analysed using the programs DNASTAR, MEGA6, and DnaSP 5.10.00. Genetic diversity and natural selection of the global *pfmsp*-*3* were also comparatively analysed.

**Results:**

Myanmar *pfmsp*-*3* displayed 2 different alleles, 3D7 and K1. The 3D7 allelic type was predominant in the population, but genetic polymorphism was less diverse than for the K1 allelic type. Polymorphic characters in both allelic types were caused by amino acid substitutions, insertions, and deletions. Amino acid substitutions were mainly occurred at the alanine heptad repeat domains, whereas most insertions and deletions were found at the glutamate rich domain. Overall patterns of amino acid polymorphisms detected in Myanmar *pfmsp*-*3* were similar in the global *pfmsp*-*3* population, but novel amino acid changes were observed in Myanmar *pfmsp*-*3* with low frequencies. Complicated patterns of natural selection and recombination events were predicted in the global *pfmsp*-*3*, which may act as major driving forces to maintain and generate genetic diversity of the global *pfmsp*-*3* population.

**Conclusion:**

Global *pfmsp*-*3* revealed genetic polymorphisms, suggesting that the functional and structural consequences of the polymorphisms should be considered in designing a vaccine based on PfMSP-3. Further examination of genetic diversity of *pfmsp*-*3* in the global *P. falciparum* population is necessary to gain in-depth insight for the population structure and evolutionary aspect of global *pfmsp*-*3*.

## Background

*Plasmodium falciparum* merozoite surface protein-3 (PfMSP-3) is a protein mainly expressed at the schizont stage of the parasite. This protein is initially synthesized as a 62 kDa precursor and then is subsequently processed into a mature protein with an approximate size of 44–48 kDa via removal of the N-terminal region in the parasitophorous vacuole [1, 2]. PfMSP-3 plays critical roles in erythrocyte binding of the parasite and protecting the parasite against toxic haem that is inevitably produced by haemoglobin hydrolysis [3]. It is also reported that PfMSP-3 is a target of host immune responses. PfMSP-3 induced naturally acquired immunity by producing antibodies that mediate antibody-dependent cellular inhibition of the parasite [3]. Immunization of PfMSP-3 induced protective immunity in animal models [4, 5]. Moreover, anti-PfMSP-3 antibodies suppressed the growth of *P. falciparum* in vitro in the presence of blood monocytes [2, 6]. Vaccines formulated with PfMSP-3 alone or in combination with other vaccine candidate antigens have been designed and their effectiveness in inducing production of cytophilic antibodies that could inhibit *P. falciparum* erythrocytic growth has been evaluated [7, 8]. GMZ2, the vaccine formulated with a chimeric antigen constructed with the C-terminal region of PfMSP-3 and the N-terminal region of glutamate rich protein (GLURP), was well tolerated and immunogenic, and elicited high levels of functional antibodies suppressing multiplication of the parasite [9–12]. The essential biological functions of PfMSP-3 during parasite survival and the accumulated evidence that specific antibodies for PfMSP-3 are effective in parasite killing and protection against clinical malaria suggest that this protein as a promising vaccine candidate antigen.

Genetic polymorphisms in vaccine candidate antigens of *P. falciparum* are great hurdles to develop effective vaccines since they could generate a variant-specific immune response that is less effective against parasites with other genetic variants. Like other vaccine candidate antigens, the genetic diversity of *pfmsp*-*3* was also reported in natural parasite populations [13–16]. The *pfmsp*-*3* consists of 3 separated regions: the N-terminal region containing four alanine heptad repeats (AHR) having the AXXXAXXX motif, the glutamate rich domain, and the C-terminal region containing a leucine zipper motif [17–20]. The gene is classified into 2 different alleles, 3D7 and K1, based on the difference in size of the AHR at the N-terminal region [17]. It has been proposed that the N-terminal region was polymorphic, whereas the C-terminal region was relatively well conserved in *P. falciparum* populations in several countries including Iran [14], Thailand [13, 15], Cameroon, Republic of Congo, Burkina Faso, Ghana, and Senegal [16]. In-depth understanding of the population genetic structure of *pfmsp*-*3* in the natural *P. falciparum* population is important not only for evaluating the genetic nature of the vaccine candidate antigen, but also for advancing effective vaccine development.

However, compared to other vaccine candidate antigens, fewer studies have been done to understand the genetic polymorphisms of *pfmsp*-*3* in the natural *P. falciparum* population. In this study, the genetic diversity and natural selection of *pfmsp*-*3* in Myanmar *P. falciparum* isolates were analysed. Comparative analysis of global *pfmsp*-*3* was also performed to gain in-depth understanding of the genetic make-up of the gene in the global *P. falciparum* population.

## Methods

### Blood samples and extraction of genomic DNA

Seventy-two blood samples were obtained from *P. falciparum* infected symptomatic patients who live in villages located in Naung Cho and Pyin Oo Lwin, Myanmar in 2015 (Additional file 1: Fig. S1). *P. falciparum* infection was confirmed by Giemsa-stained thick and thin blood smear examination. All *P. falciparum* positive samples were further confirmed by polymerase chain reaction (PCR) targeting 18S ribosomal RNA (rRNA) gene [21]. Finger-prick blood samples were collected from the patients prior to drug treatment, air-dried, and stored in individual sealed plastic bag at ambient temperature until use. Genomic DNA was extracted from the blood filters using the QIAamp DNA Blood Kit (Qiagen, Hilden, Germany) following the manufacturer’s instructions. Written informed consent was obtained from all patients before blood collection. The study protocol was approved by either the Ethics committee of the Ministry of Health, Myanmar (97/Ethics 2015) and the Biomedical Research Ethics Review Board of Inha University School of Medicine, Republic of Korea (INHA 15-013).

### Amplification and sequencing analysis of *pfmsp*-*3*

The full-length *pfmsp*-*3* was amplified by a nested PCR method. The primers used in this study were designed as described previously [13]. The thermal cycling parameters for primary and nested PCRs were as follows: one cycle of initial denaturation at 95 °C for 5 min, 25 cycles of 94 °C for 1 min, annealing at 57 °C for 2 min and extension at 72 °C for 2 min, followed by a final extension at 72 °C for 5 min. *Ex Taq* DNA polymerase (Takara, Otsu, Japan) with proof- reading activity was used in all PCR amplification steps to minimize the nucleotide mis-incorporation. PCR products were resolved on a 1.2% agarose gel, and were visualized under ultraviolet (UV). Each PCR product was purified from the gel, and cloned into the T&A vector (Real Biotech Corporation, Banqiao City, Taiwan). Ligation mixture was transformed into *Escherichia coli* DH5α competent cells, and positive clones with appropriate insert were selected by colony PCR. The nucleotide sequence of cloned insert was analysed by automatic DNA sequencing with M13 forward and M13 reverse primers. Plasmids from at least two independent clones from each transformation mixture were sequenced in both directions to verify the sequence accuracy. The nucleotide sequences reported in this study have been deposited in the GenBank database under the accession numbers MN787600-MN787671.

### Analyses of sequence polymorphism and genetic diversity of Myanmar *pfmsp*-*3*

The nucleotide and deduced amino acid sequences of *pfmsp*-*3* were analysed using EditSeq and SeqMan in the DNASTAR package (DNASTAR, Madison, WI, USA). The *pfmsp*-*3* sequences of 3D7 (XM_001347593) and K1 (U08851) were used as reference sequences. The value of singleton variable sites, parsimony informative sites, total number of mutations, segregating sites (S), average number pair-wise nucleotide difference (*K*), haplotype diversity (Hd), and nucleotide diversity (π) were calculated using DnaSP version 5.10.00 [22]. The π was also measured on sliding window plot of 10 bases with a step size of 5 bp to estimate the stepwise diversity across the sequences. The rate of synonymous (dS) and non-synonymous (dN) substitutions were estimated and compared by the Z-test (*P* < 0.05) in order to test the null hypothesis of strict neutrality of *pfmsp*-*3* in MEGA6 program [23] using the Nei and Gojobori’s method [24] with the Juke and Cantor correction (1000 bootstrap replications). Tajima’s D test [25], and Fu and Li’s D and F statistics [26] were performed to evaluate the neutral theory of natural selection using DnaSP version 5.10.00 [22]. The Tajima’s D values were also calculated on sliding window plot of 10 bases with a step size 5 bp to estimate the natural selection parameter across the gene.

### Genetic diversity of *pfmsp*-*3* among the global *P. falciparum* isolates

The genetic diversity of global *pfmsp*-*3* from different *P. falciparum* populations was analysed. The *pfmsp*-*3* sequences included in this study were from Thailand (AM161544-AM161593), India (HM568669-HM568724), Kenya (KU527019-KU527058), and Nigeria (AM161594-AM161644). These sequences covered full-length or partial of *pfmsp*-*3*. Genetic polymorphism and tests of neutrality of each population were analysed or calculated using DNASTAR package, DnaSP version 5.10.00 [22] and MEGA6 [23] as describe above. A logo plot was constructed for each *pfmsp*-*3* population to analyse the polymorphic patterns in the B cell epitopes using the WebLogo program (https://weblogo.berkeley.edu/logo.cgi).

## Results

### Sequence polymorphisms of Myanmar *pfmsp*-*3*

**Fig. 1:**
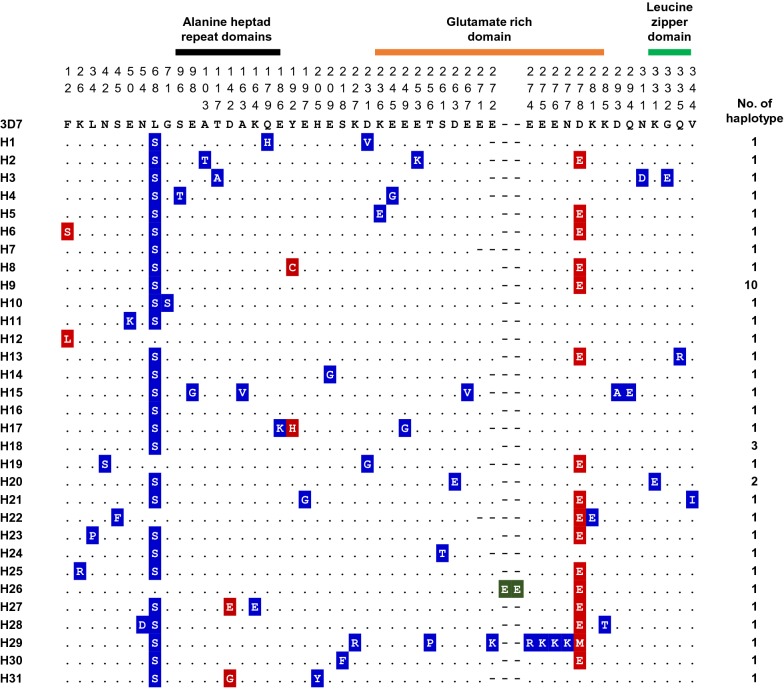
Sequence polymorphisms of 3D7 allelic types of Myanmar *pfmsp*-*3*. A total of 31 haplotypes of 3D7 allelic types were detected in Myanmar *pfmsp*-*3*. The dots present amino acid residues identical to the reference sequence of 3D7 (XM_001347593). Dashes are gaps introduced to maximize the alignment. Numbers above the alignment are amino acid positions with reference to the 3D7 sequence. The di-morphic and tri-morphic amino acid changes at particular positions are shaded by blue and red, respectively. The insertion found in Myanmar *pfmsp*-*3* is marked by green. The number of each haplotype found in Myanmar *pfmsp*-*3* is indicated at right

**Fig. 2:**
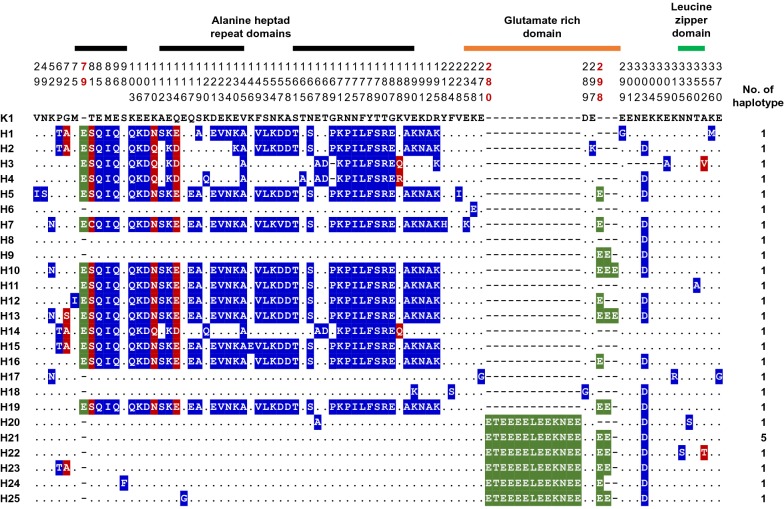
Amino acid polymorphisms of K1 allelic types of Myanmar *pfmsp*-*3*. A total of 25 haplotypes of K1 allelic types were detected in Myanmar *pfmsp*-*3*. The dots present residues identical to the reference sequence of K1 (U08851). Dashes are gaps introduced to maximize the alignment. Numbers above the alignment are amino acid positions with reference to the K1 sequence. The di-morphic and tri-morphic amino acid changes at particular positions are shaded by blue and red, respectively. The insertions found in Myanmar *pfmsp*-*3* are marked by green. The amino acid positions with insertion are numbered with red bold. The number of each haplotype found in Myanmar *pfmsp*-*3* is indicated at right

Seventy-two *pfmsp*-*3* were successfully amplified from Myanmar *P. falciparum* isolates. Sequence alignment analysis of the sequences suggested that they were classified into 2 allelic types, 3D7 type (*n* = 43) and K1 type (*n* = 29). For the 3D7 allelic types, 164 single nucleotide polymorphisms (SNPs) were identified at 87 polymorphic sites, in which 122 SNPs were non-synonymous mutations, resulting in amino acid substitutions at 49 positions. Four amino acid positions showed tri-morphic changes (F12L/S, D142E/G, Y192C/H, and D278E/M) and the others were di-morphic amino acid changes. The most predominant amino acid substitutions identified in Myanmar *pfmsp*-*3* were L68S and D278E/M, which accounted for 90.7% and 58.1% in frequency, respectively. The other amino acid changes occurred at very low proportions. Amino acid changes at 25 positions (G71S, E98G, T117A, D142E/G, A163V, Y192C/H, K236E, E245G, E249G, E253K, S261T, D263E, E267V, E274R, E275K, E276K, N277K, D278M, K281E, K285T, N311D, K331E, G332E, Q335R, and V344I) were novel ones that have not been reported previously. The glutamate rich domain of Myanmar *pfmsp*-*3* was varied because of different repeat numbers of glutamate residues, ranging from 4 to 8 repeats. Deletions of E271 and/or E272 were identified in 12 distinct haplotypes and insertion of 2 additional glutamates was found in haplotype 26. Based on these polymorphic patterns, the 3D7 allelic types of Myanmar *pfmsp*-*3* were further classified into 31 distinct haplotypes, in which haplotype 9 had the highest prevalence (23.3%) (Fig. 1). In the K1 allelic types, a total of 25 distinct haplotypes were identified. Haplotype 21 was the most prevalent, accounting for 17.2%. Sequence analysis revealed that there were 944 SNPs at 117 polymorphic sites, among which 576 SNPs were non-synonymous ones leading to amino acid substitutions at 75 positions. Sixty-nine di-morphic and 6 tri-morphic (P72A/S, T81C/S, K110N/Q, Q114D/E, K188Q/R, A352T/V) amino acid changes were found. High frequency of a glutamate (E) insertion at position 79 was identified at AHR domains. Insertions of 13 amino acids (ETEEEELEEKNEE) and 1–3 glutamate (E) were also detected at positions 280 and 298 in the glutamate rich domain, respectively. Amino acid deletions at 6 positions (G169, E301, N302, E303, K304, and K305) were also found in the K1 allelic types of Myanmar *pfmsp*-*3* (Fig. 2).

### Sequence polymorphism and genetic diversity of global *pfmsp*-*3*

**Fig. 3:**
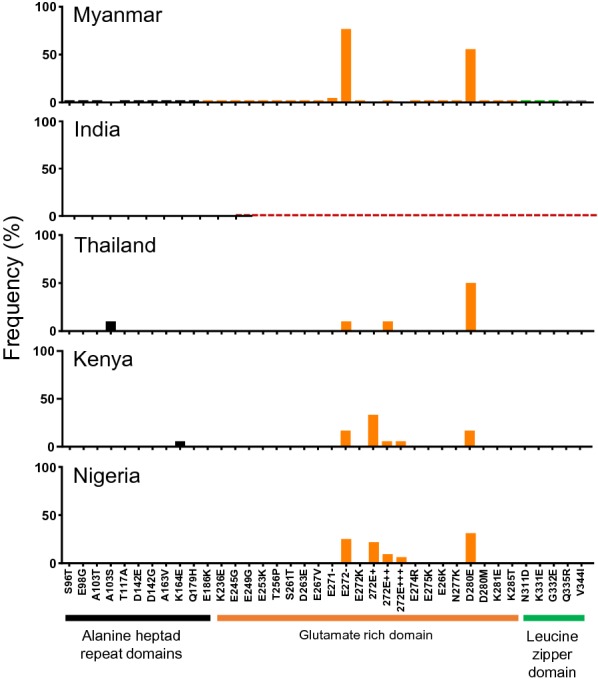
Comparison of amino acid polymorphisms in 3D7 allelic types of *pfmsp*-*3* from different geographical areas. Positions and frequencies of amino acid changes found in *pfmsp*-*3* from different countries were compared. Each domain is presented by a different color bar: alanine heptad repeat domains (black), glutamate rich domain (orange), and leucine zipper domain (green). The dotted red bar in India *pfmsp*-*3* means a missed sequence. The E271- and E272-represent deletion of an amino acid in the corresponding site. The 272E + , 272E ++, and 272E +++ indicate the numbers of inserted glutamate residues at the positions with 1, 2, and 3, respectively

**Fig. 4:**
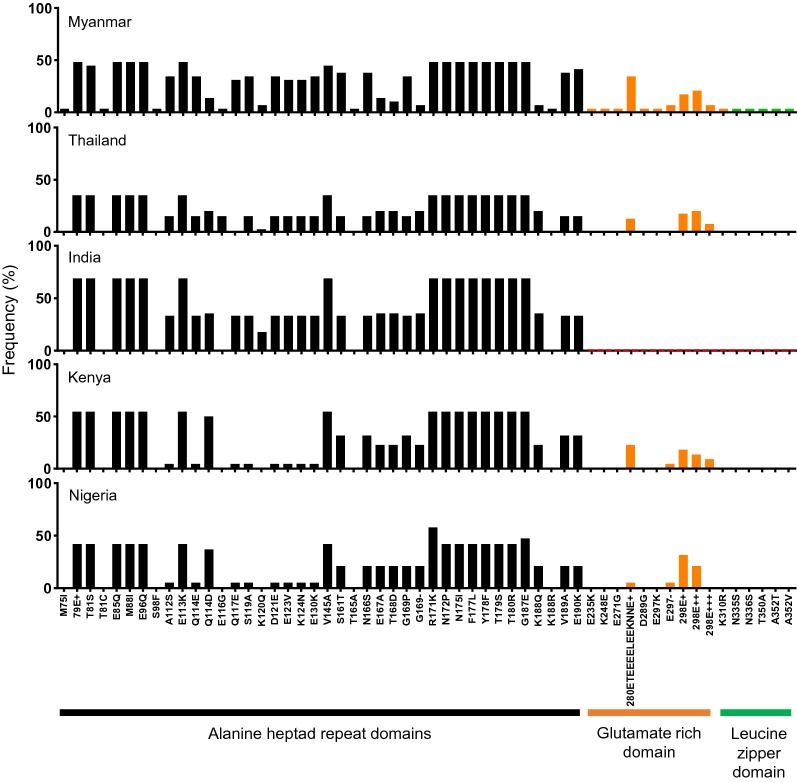
Comparison of amino acid polymorphism of K1 allelic types of *pfmsp*-*3* between Myanmar and different geographical areas. Positions and frequencies of amino acid changes found in *pfmsp*-*3* of Myanmar and other countries were compared. Each domain is presented by different color bar; alanine heptad repeat domains (black), glutamate rich domain (orange), and leucine zipper domain (green). The dotted red bar in India *pfmsp*-*3* means missed sequence. The G169- and E297- represent deletion of an amino acid in the corresponding site. E272ETEEEELEEKNNE + means insertion in the position. The 298E + , 298E ++, and 298E +++ mean the numbers of inserted glutamate residues at the positions with 1, 2, and 3, respectively

**Fig. 5:**
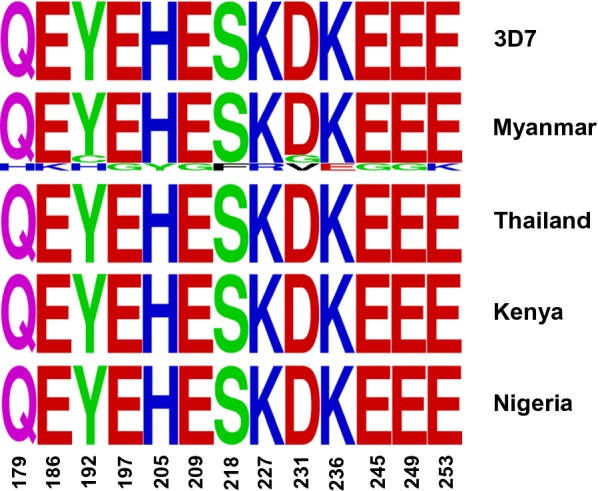
Polymorphic patterns of B-cell epitope in 3D7 allelic types of *pfmsp*-*3* from Myanmar and different *P. falciparum* isolates. A logo plot was constructed for each *pfmsp*-*3* population using the WebLogo program

Comparative analysis of genetic polymorphism of *pfmsp*-*3* from Myanmar and other different geographical areas was performed. Given the partial sequence information of *pfmsp*-*3* from other global populations, analysis focused on the 3 regions corresponding to AHR, the glutamate rich domain and the leucine zipper domain. For 3D7 allelic types, most amino acid polymorphisms were accumulated at glutamate rich domains, but the frequencies were different by country (Fig. 3). The deletion of E272 (E272-) and an amino acid change (D280E) were found in all analysed populations. Insertions of 1 to 3 glutamate residues at the position of 272 (E272 + , E272 ++, and E272 +++) were more prevalent in African *pfmsp*-*3* than in Asian *pfmsp*-*3*. The AHR and leucine zipper domains were relatively well conserved in 3D7 allelic types of global *pfmsp*-*3*. Amino acid changes at 9 positions were identified in AHR domains of global *pfmsp*-*3*, most of them only in Myanmar *pfmsp*-*3*. Only 2 amino acid changes (A103S and K164E) were found in Thailand and Kenya *pfmsp*-*3*. The five amino acid substitutions (N311D, K331E, G332E, Q335R, and V344I) in the leucine zipper domain were detected only in Myanmar *pfmsp*-*3*. Polymorphic patterns of amino acid changes were more diverse and greater in global K1 allelic types (Fig. 4). Most amino acid changes detected at AHR domains were shared by the global *pfmsp*-*3* population with high frequencies. Only five amino acid substitutions, G75I, T81C, S98F, T165A, and K188R, were uniquely identified in Myanmar *pfmsp*-*3*. Although the overall patterns of amino acid polymorphisms identified in global *pfmsp*-*3* were similar, the frequencies of A112S, Q114E, Q117E, S119A, D121E, E123V, K124M, and E130K were relatively higher in Asian *pfmsp*-*3* than in African *pfmsp*-*3*. The glutamate rich domain also showed polymorphic patterns in global *pfmsp*-*3*. Insertions of glutamate (E) at position 79, 13 amino acids (ETEEEELEEKNEE) at position 280, and glutamate (E) at the position 298 were found in *pfmsp*-*3* from Thailand, Kenya, and Nigeria. Deletion of glutamate (E) at position 297 was also identified in *pfmsp*-*3* from Kenya and Nigeria. Meanwhile, the leucine zipper domain was well-conserved in global *pfmsp*-*3*. Only 5 minor amino acid changes (N335S, N336S, T350A, A352T, and A352V) were uniquely identified in Myanmar *pfmsp*-*3*. Polymorphic patterns of the B-cell epitopes of 3D7 allelic types of global *pfmsp*-*3* were analysed. The amino acid residues constructing the B-cell epitopes were tightly conserved in global *pfmsp*-*3* allelic types (Fig. 5). However, di-morphic and tri-morphic amino acid changes at the corresponding residues were detected in Myanmar *pfmsp*-*3*.

### Genetic diversity and natural selection of Myanmar *pfmsp*-*3*

**Fig. 6:**
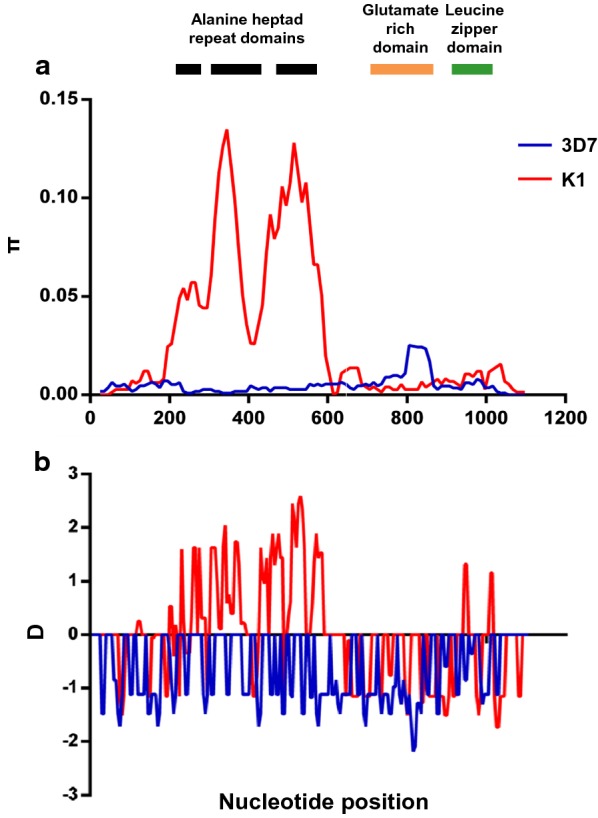
Nucleotide diversity and test of natural selection in Myanmar *pfmsp*-*3*. **a** Sliding window plot presented nucleotide diversity (π) values in Myanmar *pfmsp*-*3*. A window size of 10 bp and step size of 5 bp were used. **b** Sliding window calculation of Tajima’s D statistic was performed in Myanmar *pfmsp*-*3*. A window length of 10 bp and step size of 5 bp were used. Each domain is presented by different color bar on the graphs: alanine heptad repeat domains (black), glutamate rich domain (orange), and leucine zipper domain (green)

The genetic diversity and tests of neutrality of Myanmar *pfmsp*-*3* were analysed. The average number of nucleotide differences (*K*) for 3D7 and K1 allelic types were 5.30 and 33.22, respectively. The 3D7 and K1 allelic types showed similar values of haplotype diversity (Hd, 0.990 ± 0.009 for 3D7 and 0.990 ± 0.013 for K1), but nucleotide diversity (π) of K1 allelic types (0.0297 ± 0.0019) was greater than that for 3D7 allelic types (0.0050 ± 0.0006) (Table 1). The sliding window plot of π across Myanmar *pfmsp*-*3* revealed that the highest values of π were identified at AHR domains in the K1 allelic type. In contrast, the highest values of π were detected at the glutamate rich domain for the 3D7 type. The leucine zipper domains showed low values of π in both allelic types of Myanmar *pfmsp*-*3* (Fig. 6a). The dN–dS values of Myanmar *pfmsp*-*3* were negative for both 3D7 (− 0.007 ± 0.002) and K1 (− 0.016 ± 0.009), suggesting that both allelic types of Myanmar *pfmsp*-*3* were affected by negative natural selection. The value of Tajima’s D for the 3D7 allelic types was negative (− 2.692, *P* < 0.001), suggesting that 3D7 allelic types of Myanmar *pfmsp*-*3* were under purifying selection. Meanwhile, the value was positive for the K1 allelic type (0.373, P > 0.1), implying that the K1 type was under balancing selection (Table 1). The sliding window plot analysis of Tajima’s D across Myanmar *pfmsp*-*3* also indicated that the values were negative across the entire gene for the 3D7 allelic types, whereas positive values of Tajima’s D were predicted at AHR domains and the leucine rich region of K1 allelic types (Fig. 6b).

**Table 1:** Genetic polymorphism and tests of neutrality of *pfmsp*-*3* in global *P. falciparum* isolates

Country	Singleton variable sites	Parsimony informative sites	Total no. of mutations	S	*K*	H	Hd ± SD	π ± SD	dN–dS	Tajima’s D (*P* value)	Fu and Li’s D (*P* value)	Fu and Li’s F (*P* value)
3D7												
Myanmar (*n *= 43)	77	10	90	87	5.30	37	0.990 ± 0.009	0.0050 ± 0.0006	− 0.007 ± 0.002	− 2.692 (*P* < 0.001)	− 5.616 (*P* < 0.02)	− 5.438 (*P* < 0.02)
Kenya (*n *= 18)	4	2	6	6	1.11	6	0.647 ± 0.095	0.0030 ± 0.0010	− 0.003 ± 0.006	− 1.208 (*P* > 0.1)	− 1.468 (*P* > 0.1)	− 1.607 (*P* > 0.1)
Nigeria (*n *= 32)	3	8	11	11	2.98	14	0.929 ± 0.020	0.0030 ± 0.0004	− 0.012 ± 0.006	0.289 (*P* > 0.1)	− 0.092 (*P* > 0.1)	0.031 (*P* > 0.1)
Thailand (*n *= 10)	1	5	6	6	2.69	5	0.844 ± 0.080	0.0030 ± 0.0005	− 0.008 ± 0.005	1.10787 (*P* > 0.10)	0.77491 (*P* > 0.10)	0.96107 (*P* > 0.10)
K1												
Myanmar (*n* = 29)	47	68	119	115	33.22	26	0.990 ± 0.013	0.0297 ± 0.0019	− 0.016 ± 0.009	0.373 (*P* > 0.10)	− 1.074 (*P* > 0.10)	− 0.702 (*P* > 0.10)
Kenya (*n* = 22)	11	44	57	55	22.75	6	0.736 ± 0.070	0.0420 ± 0.0035	0.014 ± 0.014	1.805 (0.05 < *P* < 0.10)	0.471 (*P* > 0.10)	1.031 (*P* > 0.10)
Nigeria (*n* = 19)	8	62	74	70	26.63	13	0.906 ± 0.060	0.0280 ± 0.0034	− 0.005 ± 0.010	1.063 (*P* > 0.10)	1.042 (*P* > 0.10)	1.220 (*P* > 0.10)
Thailand (*n* = 40)	2	64	68	66	22.80	10	0.810 ± 0.039	0.0230 ± 0.0034	− 0.006 ± 0.008	1.540 (*P* > 0.1)	1.727 (*P* < 0.02)	1.976 (*P* < 0.02)

*n* number of sequences analysed, S number of Segregating sites, *K* average number of nucleotide differences, H number of Haplotypes, Hd haplotype diversity, π observed average pairwise nucleotide diversity, dN, rate of non-synonymous mutations, dS rate of synonymous mutations, SD standard deviation

### Genetic diversity and natural selection of global *pfmsp*-*3*

Influence of natural selection on the genetic diversity of the global *pfmsp*-*3* populations was analysed. Nucleotide diversity and patterns of natural selection predicted in 3D7 and K1 allelic types were different by country (Table 1). In the 3D7 allelic types, the values of nucleotide diversity (π) were similar for the *pfmsp*-*3* of each country, ranging from 0.0030 to 0.0050. The dN–dS value was negative for all countries analysed, indicating that negative natural selection may occur in global *pfmsp*-*3*. However, the value of Tajima’s D was different by country: negative for Myanmar and Kenya, but positive for Nigeria and Thailand. The values of Fu and Li’s D and F for each country also showed a similar trend of negative or positive values. Meanwhile, more complex patterns of natural selection were predicted in K1 allelic types (Table 1). The values of nucleotide diversity (π) of K1 allelic types were greater than those of 3D7 allelic types in all countries analysed. The values ranged from 0.0230 to 0.0420. The value of dN–dS was positive in Kenya, but in other countries including Myanmar, Nigeria, and Thailand was negative. Unlike the 3D7 allelic types, the values of Tajima’s D of all countries were positive, indicating they were under balancing selection. The values of Fu and Li’s D and F were also positive in all three countries except for Myanmar. These collectively suggested that the overall genetic diversity in the K1 allelic types was greater than in the 3D7 allelic types in global *pfmsp*-*3*. Both allelic types of global *pfmsp*-*3* may be affected by natural selection, but the direction of natural selection differed by country.

### Linkage disequilibrium and recombination events in Myanmar *pfmsp*-*3*

**Fig. 7:**
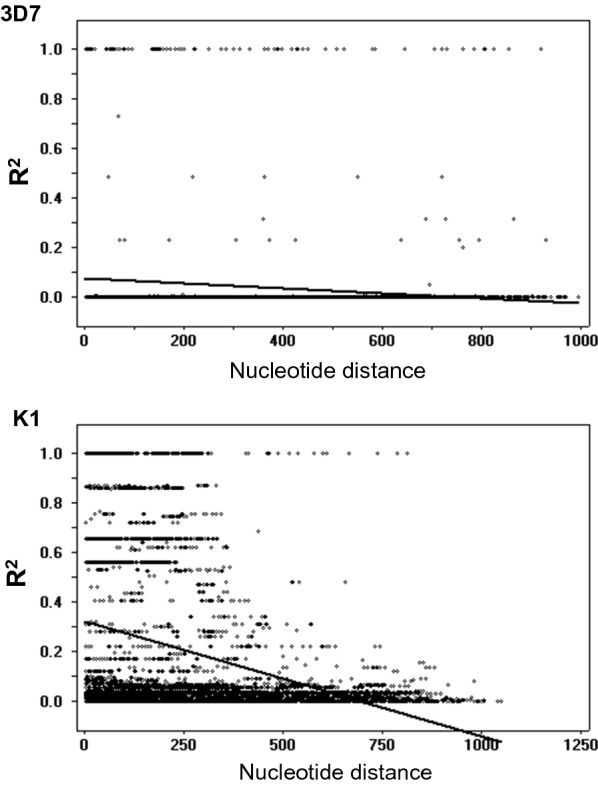
The linkage disequilibrium (LD) of Myanmar *pfmsp*-*3*. The LD plots show non-random associations between nucleotide variations in Myanmar *pfmsp*-*3* at different polymorphic sites. The R^2^ values are plotted against the nucleotide distance with two-tailed Fisher’s exact test of significance

The value for the minimum number of recombination events between adjacent polymorphic sites (Rm) for the 3D7 allelic types of Myanmar *pfmsp*-*3* was 1 (Table 2). Potential recombination sites were predicted at the positions between 203 and 906. In the K1 allelic types, the Rm value was 10, in which recombination events were predicted at the positions 121-205, 219-231, 231-333, 345-387, 420-437, 441-505, 505-515, 563-571, 571-587, and 657-1026 (Table 2). The LD index R^2^ plots for both 3D7 and K1 allelic types of Myanmar *pfmsp*-*3* declined across the analysed regions, suggesting that intragenic recombination could be a factor contributing to the genetic diversity of *pfmsp*-*3* found in Myanmar *P. falciparum* population (Fig. 7).

**Table 2:** Recombination event in Myanmar *pfmsp*-*3*

Allelic type	*n*	Ra	Rb	Rm
3D7	43	0.0171	18.2	1
K1	29	0.0023	3.2	10

## Discussion

PfMSP-3 is one of the leading vaccine candidates for malaria vaccine development, but its genetic makeup and evolutionary aspect in the global *P. falciparum* population are less understood that are other major vaccine candidate antigens, such as MSP-1, MSP-2, apical membrane antigen-1 (AMA-1), and circumsporozoite surface protein (CSP) of the parasite. In this study, genetic diversity and natural selection of Myanmar *pfmsp*-*3* were analysed. Two allelic types of *pfmsp*-*3*, 3D7 and K1, were identified in Myanmar *pfmsp*-*3* with the predominance of the 3D7 allelic type (59.7%). This is consistent with other reports that 3D7 allelic types are more prevalent in Peru [27], East Republic of Congo and Burkina Faso [16], and the Thailand-Laos border [15]. Meanwhile, the recombinant allelic type, which was reported in *pfmsp*-*3* from Iran and Africa countries with a very low frequency [14, 16], was not identified in Myanmar *pfmsp*-*3*. The lack of a recombinant allelic type may result from the large sequence difference between the two dimorphic alleles, which impedes recombination during meiosis. Otherwise, the sequence dimorphism among *pfmsp*-*3* may be strictly maintained in the population by balancing selection [28, 29]. However, recombination between and among the same allelic type of Myanmar *pfmsp*-*3* is likely to occur, considering the Rm value in each allelic type. Particularly, the K1 allelic type of Myanmar *pfmsp*-*3* revealed many recombination events, indicating that active intragenic recombination is occurring in the population.

Both 3D7 and K1 allelic types of Myanmar *pfmsp*-*3* revealed polymorphic characteristics caused by amino acid substitutions, insertions, and deletions. Amino acid substitutions mainly occurred at the AHR domain in both allelic types of Myanmar *pfmsp*-*3*. Meanwhile, insertions and deletions were found at the glutamate rich domain. The major amino acid substitutions and insertions/deletions found in both allelic types of Myanmar *pfmsp*-*3* were shared by global *pfmsp*-*3*, but their frequencies differed by geographical origin. Interestingly, novel amino acid changes that were not reported previously for either allelic type of Myanmar *pfmsp*-*3*, which renders genetic diversity of Myanmar *pfmsp*-*3* more complex; 31 and 25 haplotypes for the 3D7 and K1 allelic types, respectively. Although incidence of malaria has been remarkably decreased in Myanmar in recent years [30], it seems likely that much genetic diversity is still maintained in Myanmar *P. falciparum* population. Regardless of the declining population in recent years, substantial levels of genetic heterogeneities in *pfmsp*-*1*, *pfmsp*-*2*, *pfama*-*1*, and *pfcsp* have been reported in the Myanmar *P. falciparum* population [31–34]. The reason for this is not clearly demonstrated yet and therefore further study to understand this phenomenon would be necessary.

Despite different rates of amino acid changes and insertions/deletions in *pfmsp*-*3* from each country, the overall patterns of amino acid polymorphisms were similar in the global *pfmsp*-*3* population. Major amino acid changes and insertions/deletions found in both 3D7 and K1 allelic types were observed at the AHR and glutamate rich domains. The repeats of the AXXAXXX motif, which plays a role in maintaining and forming an α-helical secondary structure of PfMSP-3 [19], were highly conserved in global *pfmsp*-*3* regardless of diversity within and flanking the ARH. The B-cell epitopes of 3D7 allelic types were also well conserved in the global *pfmsp*-*3* population. The N-terminal region of *pfmsp*-*3* including the B-cell epitopes is a target of naturally acquired immunity. In particular, the regions corresponding to amino acids 21 to 238 not only is an important immunogen to produce a protective immune response but also is involved in the interaction with PfMSP-1 [35]. The prevalence of opsonizing antibodies against the N-terminal region rather than the C-terminal region of *pfmsp*-*3* also suggests a critical role of the N-terminal region as a target of opsonizing antibodies. Therefore, the N-terminal region of *pfmsp*-*3* may represent an attractive candidate for formulation of a PfMSP-3-based vaccine. Meanwhile, the leucine zipper domain was relatively well conserved in global *pfmsp*-*3*. The limited polymorphic pattern of the C-terminal region, especially in the leucine zipper domain, may result from its crucial function in the formation of protein oligomerization [3, 36]. Moreover, the C-terminal region of *pfmsp*-*3* plays an essential function in the invasion process of merozoite by mediating specific binding with erythrocytes [37].

The dN–dS values for 3D7 allelic types of global *pfmsp*-*3* were negative, implying that negative selection might act in the gene. Meanwhile, the values of Tajima’s D and Fu and Li’s D and F revealed complicated patterns that were different by country. These suggest that a complicated natural selection may act on 3D7 allelic types of the global *pfmsp*-*3*, in which either positive selection or purifying selection might have occurred in the population, depending on the geographical origin. As expected, overall nucleotide diversity in K1 allelic types was greater than that in 3D7 allelic types in the global isolates. The positive values of Tajima’s D indicated that K1 allelic types of global *pfmsp*-*3* evolved under balancing selection. Amino acid substitutions, insertion or deletions may be the main pressure to generate genetic diversity of the allelic types. High recombination parameters also support the notion that recombination may be one of a force contributing to generate genetic diversity of K1 allelic types of global *pfmsp*-*3*.

## Conclusion

Myanmar *pfmsp*-*3* showed genetic polymorphisms. Two different alleles, 3D7 and K1, were detected in Myanmar *pfmsp*-*3*, with a higher prevalence of 3D7 allelic types. Overall patterns of nucleotide diversity and distributions of amino acid changes and insertions/deletions found in Myanmar *pfmsp*-*3* were similar to those from global *pfmsp*-*3*, even though minute regional differences were also identified between and among each population. Complicated patterns of natural selection and recombination events were predicted in the global *pfmsp*-*3*, which may act as major driving forces to maintain and generate genetic diversity of the global *pfmsp*-*3* population. These results suggest that the functional consequences of the polymorphism should be considered for development of a vaccine based on PfMSP-3. This study provides in-depth insight into the genetic nature and population structure of global *pfmsp*-*3*. Considering that rather few global *pfmsp*-*3* sequences were currently available, further examination of the genetic diversity and evolutionary aspect of *pfmsp*-*3* with more global *pfmsp*-*3* sequences would be necessary to better understand the genetic make-up of *pfmsp*-*3* in the natural *P. falciparum* population.

## Supplementary information

**Additional file 1: Fig. S1.** Map of study site. The blood samples were collected from patients who infected *P. falciparum*. Community-based survey was conducted in 3 villages in Pyin Oo Lwin, and Naung Cho, Upper Myanmar in 2015.

## Data Availability

The data supporting the conclusions of this article are provided within the article and its additional files. The original datasets analysed in this current study are available from the corresponding author upon request. The nucleotide sequences reported in this study have been deposited in the GenBank database under the accession numbers MN787600-MN787671.

## Abbreviations

AHR: Alanine heptad repeat
dS: Synonymous substitution
dN: Non-synonymous substitution
GLURP: Glutamate rich protein
Hd: Haplotype diversity
*K*: Average number pair-wise nucleotide difference
PfMSP-3: *Plasmodium falciparum* merozoite surface protein-3
PCR: Polymerase chain reaction
rRNA: Ribosomal RNA
SPAM: Secreted polymorphic antigen associated with merozoites
S: Segregating sites
UV: Ultraviolet
π: Nucleotide diversity
