# Supplementary figures and images for: Genetic polymorphism of merozoite surface protein-3 in Myanmar Plasmodium falciparum field isolates
Source: Malar J. 2020 May 19;19:184. doi: 10.1186/s12936-020-03256-y (PMC7235555; doi:10.1186/s12936-020-03256-y)

## Slide 1
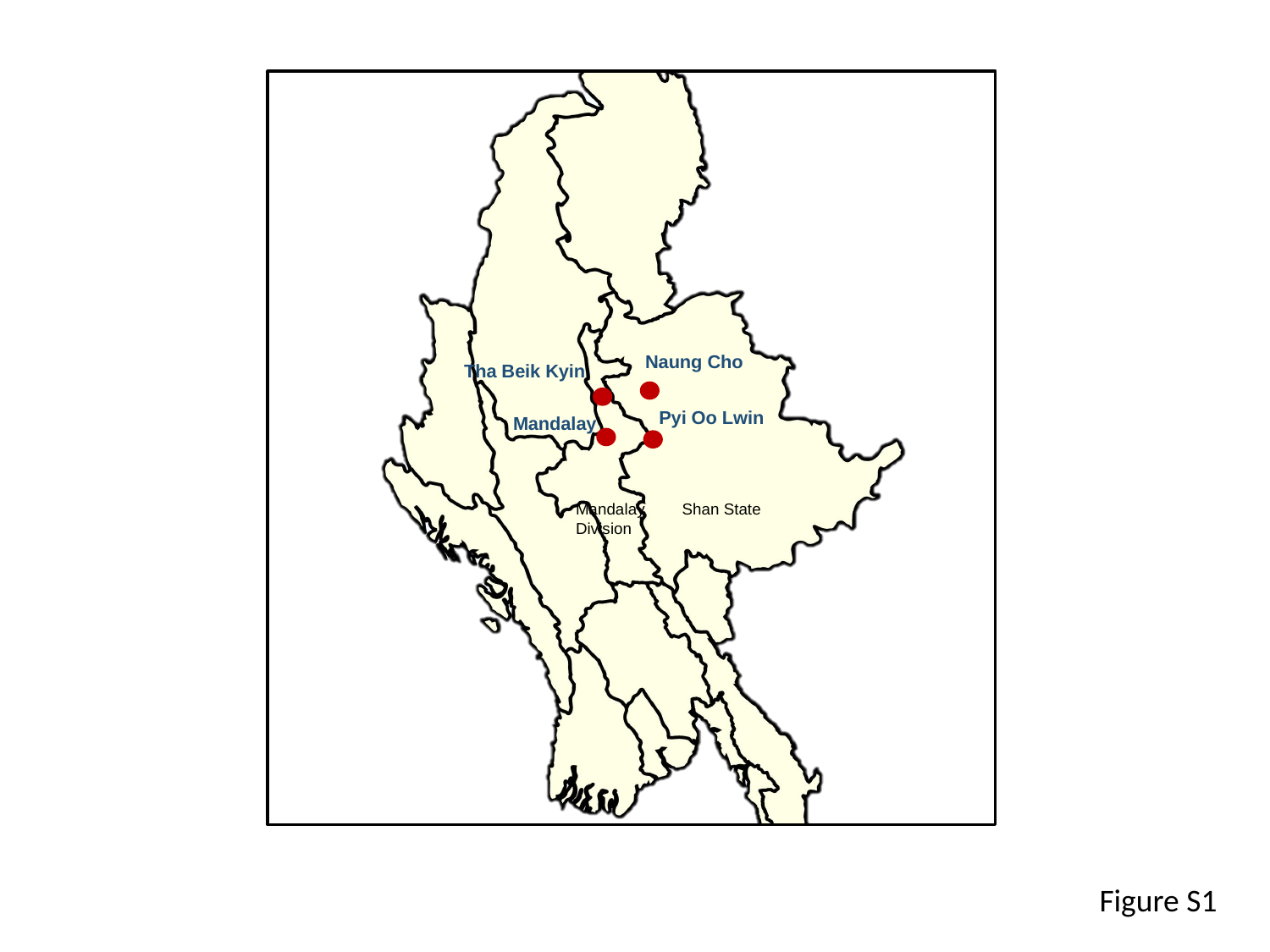

Naung Cho
Pyi Oo Lwin
Shan State
Mandalay Division
Tha Beik Kyin
Mandalay
Figure S1

Supplement: Supplementary file 1 — Additional file 1: Fig. S1. Map of study site. The blood samples were collected from patients who infected P. falciparum. Community-based survey was conducted in 3 villages in Pyin Oo Lwin, and Naung Cho, Upper Myanmar in 2015. [file 12936_2020_3256_MOESM1_ESM.pptx]
